# Post-Rhinoplasty Acne, Frequency and Role Players

**DOI:** 10.22038/ijorl.2020.41593.2358

**Published:** 2020-09

**Authors:** Mehdi Bakhshaee, Mehdi Poursadegh, Majid Irani, Mohammad Javad Yazdanpanah, Mohammad Reza Fayyazi Bordbar, Bashir Rasoulian

**Affiliations:** 1 *Sinus and Surgical Endoscopic Research Center, Mashhad University of Medical Sciences, Mashhad, Iran.*; 2 *Department of Otorhinolaryngology-Head & Neck Surgery, Mashhad University of Medical Sciences, Mashhad, Iran. *; 3 *Cutaneous Leishmaniasis Research Center, Mashhad University of Medical Sciences, Mashhad, Iran* *.*; 4 *Psychiatry and Behavioral Sciences Research Center, Mashhad University of Medical Sciences, Mashhad, Iran.*

**Keywords:** Acne, Complication, Psychological status, Rhinoplasty

## Abstract

**Introduction:**

Acne is a common complain among post rhinoplasty patients. While rhinoplasty is done for aesthetic reasons and acne expressively affects the individual’s appearance, we aimed to study its incidence and role players.

**Materials and Methods:**

A descriptive cross-sectional study was performed on 152 (143 females and 9 males) patients admitted for rhinoplasty during January 2016 to March 2017. Patients were examined by a dermatologist prior to surgery and 7, 30 and 90 days after rhinoplasty using the Global Acne Grading System and responded to a list of questions on the probable risk factors of acne. Psychological status was examined by the perceived stress scale-14 and the Hospital Anxiety and Depression Scale.

**Results:**

The patients' mean age was 28.9±3.82 yrs. Mild and moderate acne were observed in 21.7% (n=33) of the cases in the preoperative visit. The incidence of mild and moderate acne was 36.1%, 42.8% and 23% after 7, 30 and 90 days of surgery, respectively. One week after rhinoplasty, acne manifested in 14.9% of cases with no history of acne. Mean age significantly differed between those with and without post-surgical acne at all post-surgical visits (P> 0.001 and P=0.001 and P=0.015, respectively). Hospital anxiety and depression and perceived stress levels were significantly higher in patients who presented with acne on the first post-surgical visit compared to those with no acne presentations (P=0.04 and P=0.02, respectively).

**Conclusion:**

External psychological stress may be the main role player in post-rhinoplasty acne. Consultation or referral of patients to an experienced psychologist is highly recommended for a better outcome and fewer complications.

## Introduction

Acne vulgaris is known as the most prevalent skin disorder in adolescents and young adults (1). Around 80% of the world population experience acne at least once during their lifetime (2,3). Hair follicles colonization by Propionibacterium acnes and androgen induced increase in sebum formation, result in a chronic inflammation of the pilosebaceous unit which is known as acne vulgaris (4). It has previously been observed that diet has a major role in the development and severity of acne. Factors such as milk consumption, chocolate intake, omega-6 fatty acids, omega-3 fatty acids and serum zinc levels are found to be influencing the acne incidence (1,4-6). Cosmetic exposure seems to also have a correlation with acne intensity (3). Post inflammatory hyperpigmentation and facial scarring are known as the possible complications (4,7). Such complications and the lesions are believed to be responsible for the psychosocial impact such as social isolation, shame, lower levels of quality of life, altered self-esteem and depression (3,7,8).

Rhinoplasty describes a set of operative manipulations that are used to modify the aesthetic appearance and functional properties of the nose. Based on the American Society of Plastic Surgeons and the American Society of Aesthetic Plastic Surgeons annual statistics in 2016 (9,10), around 148000 to 223000 rhinoplasty surgical procedures were performed in the United States. The incidence of significant post-rhinoplasty complications has been reported to range from 1.7% to 18%. Common post-rhinoplasty complications include bleeding, infection, obstruction of the nasal airway and deformities (11).

In our clinic, the incidence of acne is one of the main postoperative complains in rhinoplasty patients. To date, there are few studies which have investigated post rhinoplasty acne. In a case-control study by Sadeghi et al. the incidence of acne was significantly higher in the open rhinoplasty group compared to the closed septoplasty group (12). Nemati et al. also reported similar results in a study on 110 rhinoplasty patients and 80 septoplasty cases as the control group (13). Accordingly, higher rates of acne, seborrhea and ecchymosis in open septorhinoplasty versus the septoplasty group was observed in a study of 50 patients by Koc et al (14).

None of these studies have investigated the factrs which may be the cause of acne formation and their role. Therefore, due to the lack of studies on the incidence of acne after rhinoplasty and the leading factors, we aimed to study its incidence and the role of each possible factor.

## Materials and Methods

A descriptive cross-sectional study was performed on 151 patients (143 females and 8 males) aged between 18 to 40 years who were admitted for rhinoplasty in Ghaem and Imam Reza hospitals of Mashhad, Iran during Jan. 2016 to March 2017. Patients who consumed testosterone, progesterone, lithium, phenytoin, vitamin B2, B6, B12, epithelial growth factor inhibitors, long term steroids and any other drugs inducing acne-like lesions and also cases with any cutaneous (atopic dermatitis and contact dermatitis prior to surgery), infectious, endocrine (polycystic ovary, Cushing's syndrome, congenital adrenal hyperplasia, androgen producing tumors, etc.), autoimmune (e.g. Behcet’s syndrome, systemic lupus erythematosus and SAPHO syndrome) or other diseases causing acne or acne-like lesions were excluded.

After filling the inclusion criteria, a written informed consent was obtained from each participant. Incidence and severity of acne was observed prior to rhinoplasty and in the one week, one-month and three-month visits after surgery using the Global Acne Grading System (GAGS) which is a scoring system based on the type and count of acne lesions on the face, chest and upper back areas (15). Patients also responded to a questionnaire which was designed by a single dermatologist with considering the previous studies and in order to obtain demographic data and probable risk factors for acne incidence; it included the personal and family history of acne, smoking, body weight, sex, etc. The patients also had to fill the perceived stress scale-14 (pss-14) questionnaire under the supervision of a psychologist. This questionnaire consists of 14 questions scoring from 0 to 4. Score 19 was considered as the cut off point to assess the individuals condition (16). The third questionnaire was used to evaluate the Hospital Anxiety and Depression Scale (HADS) and was answered by the patients in all 4 visits (17).

All patients received prophylactic antibiotic therapy and a single dose of dexamethasone during surgery as part of their therapeutic regimen. They also had a nasal cast for 6 days followed by 3-month use of 3M adhesive tape on their nose up to the nasofacial sulcus. Postoperative drugs such as short-term antihistamine, acetaminophen, ibuprofen and amoxicillin were also prescribed.The collected data from all visits and questionnaires were gathered and analyzed using the SPSS software ver. 21. A P <0.05 was considered as the indicator of statistical significance.

## Results

In total, 152 patients (9 males and 143 females) were studied. Their mean age was 28.9±3.82. 78.3% had no type of acne prior to surgery whereas 21.7% had mild to moderate acnes in the pre-op visit. The prevalence of preoperative acne and its severity has been presented in Table 1.

**Table 1:** prevalence of acne before surgery

**The severity of acne**	**Percent**	**Frequency**
Without acne	%7803	119
Mild	%1907	30
Moderate	%2	3
Sever	0	0
Total	%100	152

The frequency and incidence of acne was studied at three time points: one week, one month and 3 months after surgery (Table.2,3).

**Table 2:** prevalence of acne at three periods of time

	**Severity of acne**	**Number**	**Percent**
first week after rhinoplasty	Without acne	97	%63.8
	mild	49	%32.2
	moderate	6	%3.9
	Severe	0	0
First month after rhinoplasty	Without acne	87	%57.2
	Mild	43	%28.3
	Moderate	22	%14.5
	Severe	0	0
third month after rhinoplasty	Without acne	117	%77
	Mild	30	%19.7
	Moderate	5	%3.3
	Severe	0	0

**Table 3:** Incidence of acne at three periods of time

**Incidence The First Week**	(%13)19	(%1.9)3	0	
**Incidence The First Month**	(%11)13	(%12)19	0	
**Incidence The Third Month**	0	(%1.3)2	0	
**Overall Incidence**	(%24)32	(%15.2)24	0	

Accordingly, in the first post-op week 36.1% of the studied cases had acne; the highest rate of acne was recorded one month after rhinoplasty as 42.8% whereas the lowest rate was achieved 3 months after surgery (23%). Regarding acne severity, they were mostly mild to moderate and severe acne was observed in none of the cases during the study course.

In general, 104 (68.4%) cases had a positive history of acne whereas it was negative in 48 (31.6%). In the 1st week following surgery acne appeared in 14.9% of the cases with a negative history; however, no correlation was found between the occurrence of acne with the pre-operative history based on the Chi-square test (P=0.612). According to the obtained results, no significant relationship was found between post-surgical acne and the patient's sex, skin type, history of acne, fatty diet, fast food consumption, irregular menstruation and smoking history.

The mean difference in age in the with and without acne groups at all post-surgical visits was statistically significant; the correlation is presented in Table 4.

**Table 4:** Relationship between frequency of acne and age

		**Mean age**	**SD**	**P value**
First week	Without acne	28.22	5.37	> 0.001
	With acne	24.16	5.04	
First month	Without acne	28.00	5.71	0.001
	With acne	25.08	4.98	
Third month	Without acne	27.35	5.69	0.015
	With acne	24.74	4.77	

As demonstrated in Table 5, only 29.6% of the participants were normal for hospital anxiety and depression levels; 70.4% of the rhinoplasty candidates suffered from hospital anxiety and depression disorder from which 31.6% had moderate to severe disorder.

**Table 5:** hospital anxiety and depression levels of the rhinoplasty candidates

**Hospital anxiety and Depression levels**	**Number**	**Percentage**
Normal	46	29.6
Borderline	59	38.8
Moderate	29	19.8
severe	18	11.8
Total	152	100

In addition, 103 (67.8%) of the studied cases had abnormal perceived stress levels whereas it was normal in 49 (31.5%). The perceived stress and hospital anxiety and depression levels were significantly higher in those who experienced acne in the 1st post-surgical week compared to those with no acne (P=0.02 and P=0.04, respectively). These levels showed no meaningful difference between the two groups in the next visits.

Regarding the correlation between acne appearance one week and one month after surgery and the demographic data, logistic regression analysis revealed that both age and the stress and anxiety level can affect the acne incidence; as with an increase in age the risk of acne appearance decreases. In this respect, with increased hospital stress and anxiety levels in the first post-surgical week, the risk of acne appearance increased by 2.1-2.9 times. In the first post-surgical month the risk increased by 2.1-2.6 times (Table.6). 

Moreover, the risk of acne appearance following increased perceived stress levels increased by 2.2 times in the first week and 2.3 times in the first month.

After surgery, the most common anatomic areas involved by acne was nose and after that were the left cheek, chin, right cheek, forehead and chest and back, respectively.

**Table 6:** regression test in variables of acne incidence

		**B**	**Exp (B) OR**
Regression Test Between variables in the first month	Age	-0.103	0.902
	Weight	-0.009	0.991
	Personal history	0.164	1.178
	Family history	-.322	0.725
	Regular menstruation	.294	1.342
	Cigarette	1.323	3.756
	Anxiety (mild)		2.1
	Anxiety (moderate)	0.972	2.644
	Anxiety (sever)	0.740	2.095
	Perceived Stress	0.872	2.344

## Discussion

 Complications following cosmetic procedures seem to be less acceptable for both the patient and the surgeon. On the other hand, patients who are a candidate for undergoing elective cosmetic surgeries may be more sensitive about their body appearance. The initial objective of this study was to evaluate the incidence and characteristics of post-rhinoplasty acne besides identifying its underlying risk factors. The most important clinically relevant finding was a significant correlation between the incidence of acne and the anxiety, depression and stress levels. 

 Normal levels of hospital anxiety and depression were seen in 29.6%, borderline levels in 38.8% and moderate to severe levels in 31.6% of rhinoplasty candidates; 67.8% of the patients also had abnormal perceived stress levels. Statistical analyses revealed an increase in the risk of acne following increased anxiety and stress levels. The highest incidence was recorded at 30 days after surgery.

More than 29% of patients without a history of acne demonstrated acne lesions in the post-surgical visits and the mean age was significantly lower in the acne group. In logistic regression analysis, an increased risk of incidence in the first week and first month of surgery was observed in those with advanced levels of hospital anxiety and depression whereas a decreased risk was recorded among the elder patients. Therefore, it seems that with an increase in perceived stress levels the risk of acne presentation in the first week increases.

The role of psychological stress in post-rhinoplasty acne has been discussed in very few studies to date. Some reports have indicated an increase in acne severity following this type of surgery (13,14). Coban in 2007 stated that a decrease in acne symptoms after rhinoplasty could be a sign of successful surgery by reduced levels of anxiety and neuroticism (18).

Several studies have suggested that emotional stress from external sources may cause a significant worsening in the acne condition (19,20). However, Nemati et al. reported an ignorable role for surgical stress due to a non-significant difference between their acne and control groups (13). On the other hand, as the candidates of rhinoplasty suffer from high levels of anxiety and stress, it is hypothesized that psychological stress is a possible underlying cause of acne formation or its exacerbation. Psychoimmunological and psychoendocrino- logical pathways may be the underlying mechanisms for the effect of stress and anxiety on acne. 

There is a communication between the brain and skin which passes through the psycho-immuno- endocrine- cutaneous system. Neuropeptides, interleukins, and immune system messengers are the main actors of this system which are affected by emotional stressors, psychological diseases, etc. and could lead to many skin disorders including acne vulgaris. The hypothalamic-pituitary-adrenal axis agents are a major role player among them (21). In addition, significant increase in serum cortisol level is reported in post-surgical patients and there is evidence of local secretion of corticotrophin-releasing hormones from sebocytes and dermal nerves which cannot be ignored (22,23).

 The peak incidence and severity of acne in the 30th post-surgical day visit and the subsequent decrease afterwards, could be justified by psychological adaptation with the post-surgical condition. A decrease in the anxiety level regarding the outcome of surgery is another probable reason for the remission of acne after one month (14,18).

The lower age in the acne group may be due to the higher prevalence of acne in younger individuals and a substantial decrease of prevalence in the 4th decade of life in the general population (24,25).

Regarding the type and localization, comedons are the most prevalent kind of lesions reported by other studies followed by macules and papules (26,27). Face is the most common affected site in the literature while the chest stands in the second place (26).

There are other possible role players which were not assessed in our study. Environmental factors such as residential area, climate, pollutants and factors such as race can affect the incidence and severity of acne in different societies. As skin injury can lead to skin inflammation and acne, the surgical procedures and techniques should be investigated. Moreover, the acne incidence varies in different seasons, therefore future studies are recommended to be conducted throughout a whole year to better determine the seasonal influence. The role of antibiotic prophylaxis and changes in skin microbial flora are notable as well.

Furthermore, the adhesive tapes and casts used and the lack of sufficient face washing can also lead to proliferation of propionibacterium acnes and acne vulgaris. After one month when the casts and tapes are removed and the face is more frequently washed, it is concurrent with the beginning of the reduction in acne incidence. It is noticeable that in patients presenting with acne, areas other than nasal skin such as the chest and back are also involved which opposes the hypothesis on the effect of the above mentioned factors.

Taken together, administration of corticosteroids during surgery, small study sample, unassessed factors which were listed above, an overall young-aged study population and short term follow up were the main limitations of the current study.

## Conclusion

Logistic regression analyses revealed a positive correlation between the acne incidence and hospital anxiety and depression and perceived stress levels; however, age was inversely correlated. Nevertheless, it seems that external psychological stress may be the major player in post-rhinoplasty acne.

Understanding the underlying causes and the way they act could help the surgeons and patients to have a better outcome with fewer complications. Surgeons are also expected to inform their patients about the role of stress and anxiety and help them control it. Consultation or patients' referral to an experienced psychologist in different time periods before and after surgery could also be beneficial. Accordingly, assessing the missing factors and especially the role and power of each stressor in a rhinoplasty patient besides studying the appropriate prophylactic interventions are highly recommended in future studies.
